# Effect of Folic Acid and Vitamin B_12_ on Pemetrexed Antifolate Chemotherapy in Nutrient Lung Cancer Cells

**DOI:** 10.1155/2013/389046

**Published:** 2013-07-31

**Authors:** Tsung-Ying Yang, Gee-Chen Chang, Shih-Lan Hsu, Yi-Rou Huang, Ling-Yen Chiu, Gwo-Tarng Sheu

**Affiliations:** ^1^Institute of Medicine, Chung Shan Medical University, No. 110, Sec 1, Jianguo N. Road, Taichung 402, Taiwan; ^2^Division of Chest Medicine, Department of Internal Medicine, Taichung Veterans General Hospital, No. 160, Sec 3, Chung-Kang, Taichung 407, Taiwan; ^3^Department of Medicine, School of Medicine, National Yang-Ming University, 155, Sec 2, Lih-Non Street, Shih-Pai 112, Taipei, Taiwan; ^4^Institute of Biomedical Sciences, National Chung Hsing University, No. 160, Sec 3, Chung-Kang, Taichung 407, Taiwan; ^5^Department of Education and Research, Taichung Veterans General Hospital, No. 160, Sec 3, Chung-Kang, Taichung 407, Taiwan; ^6^Department of Medical Oncology and Chest Medicine, Chung Shan Medical University Hospital, No. 110, Sec 1, Jianguo N. Road, Taichung 402, Taiwan

## Abstract

Pemetrexed (MTA) is a multitargeted antifolate drug approved for lung cancer therapy. Clinically, supplementation with high doses of folic acid (FA) and vitamin B_12_ (VB12) lowers MTA cytotoxicities. An antagonistic effect of FA/VB12 on MTA efficacy has been proposed. However, patients who receive FA/VB12 show better tolerance to MTA with improved survival. The aims of this study are to investigate the modulation of FA and VB12 on MTA drug efficacy in human nonsmall cell lung cancer (NSCLC) cell lines. The sensitivities of cells, apoptosis, and MTA-regulated proteins were characterized to determine the possible effects of high doses of FA and VB12 on MTA efficacy. MTA has the lowest efficacy under 10% serum conditions. However, supplementation with FA and VB12 individually and additively reversed the insensitivity of NSCLC cells to MTA treatment with 10% serum. The enhanced sensitivities of cells following FA/VB12 treatment were correlated with increasing apoptosis and were specific to MTA but not to 5-fluorouracil (5-FU). Enhanced sensitivity was also associated with p21^WAF1/Cip1^ expression level. Our results revealed no antagonistic effect of high doses of FA/VB12 on MTA efficacy in cancer cells grown in nutrient medium. Furthermore, these data may partially explain why supplementation of FA and VB12 resulted in better survival in MTA-treated patients.

## 1. Introduction

Pemetrexed (MTA, ALIMTA, LY231514, Eli Lilly and Company, IN, USA) is a novel antifolate drug that has been approved for first-line treatment of patients with advanced nonsquamous, nonsmall cell lung cancer (NSCLC) in combination with cisplatin and as a single agent for relapsed or chemotherapy refractory NSCLC after platinum-containing chemotherapy. Recently, in a double-blind study, maintenance MTA plus best supportive care cohort showed superior progression-free survival and overall survival when compared with placebo plus best supportive care in patients whose disease had not progressed after four cycles of platinum-based doublet induction chemotherapy [1]. Undoubtedly, MTA will continue to be used extensively in patients with NSCLC. MTA has also demonstrated clinical activity in a broad array of other solid tumors, including mesothelioma [2] and breast [3], colorectal [4], bladder [5], cervical [6], gastric [7], and pancreatic [8, 9] cancers.

MTA is a unique folate antagonist that inhibits thymidylate synthase (TS), dihydrofolate reductase (DHFR), and the purine synthetic enzyme glycinamide ribonucleotide formyltransferase (GARFT) [10, 11]. In early clinical trials, pretreatment plasma homocysteine and methylmalonic acid levels were used as markers of folic acid (FA) and vitamin B_12_ (VB12) deficiencies to predict severe MTA-induced toxicities [12]. This implies a correlation between deficiencies in FA and VB12 and toxicities of MTA. When there are insufficient amounts of FA and VB12 in the body, MTA may affect noncancerous and cancerous cells, which in turn leads to toxic effects. Supplementation with these two vitamins resulted in decreased MTA-related toxicities in patients with malignant pleural mesothelioma [13]. FA and VB12 are currently prescribed in dosages of 350–1,000 *μ*g per day and 1,000 *μ*g every 3 cycles, respectively, prior to and during treatment with MTA.

FA is a precursor of 5,10-methylene-tetrahydrofolate (5,10-CH_2_-THF) on which TS and DHFR are dependent for purine synthesis. Folates also serve as methyl donor in the synthesis of methionine. The methionine cycle that provides one-carbon moiety for cellular methylation reactions is also dependent on VB12. The methyl-THF that is derived from 5,10-CH_2_-THF provides methyl group and converts homocysteine to methionine by VB12 and methionine synthase [11, 14]. Since MTA is an antifolate drug that competes with natual folates, there is a concern that supplements of VB12 and FA may increase the pool of intracellular folates and reduce the efficacy of MTA chemotherapy, even though this phenomenon has not been observed in clinical trial [13]. Therefore, the aim of this study was to evaluate how high doses of FA and VB12 modulate MTA cytotoxicity in NSCLC cell lines that were grown without FA and VB12 deficiencies.

## 2. Materials and Methods

### 2.1. Cell Culture and Drug Treatment

Human NSCLC cell lines A549, H1299, and H460 were cultured at 37°C in RPMI 1640 medium and supplemented with 10% fetal bovine serum (FBS, GIBCO, Invitrogen, Carlsbad, CA, USA), 0.03% L-glutamine, 100 *μ*M sodium pyruvate, 0.22% sodium bicarbonate, 100 *μ*M NEAA, and 100 IU/mL penicillin/streptomycin. 

### 2.2. Drugs and Supplements

MTA was provided by Eli Lilly Corporation (Indianapolis, IN, USA). Methotrexate (MTX), 5-fluorouracil (5-FU), VB12, FA, and propidium iodide (PI) were purchased from Sigma-Aldrich (St. Louis, MO, USA). CellTiter 96 MTS Reagent Powder was purchased from Promega (Madison, WI, USA), and phenazine methosulfate (PMS) was obtained from Sigma-Aldrich. The drugs were dissolved in dimethyl sulfoxide (DMSO) (MTX, 5-FU), 1 M NaOH (FA), and ddH_2_O (MTA, VB12), respectively, and stored at −20°C. They were diluted in culture medium immediately before use.

### 2.3. Growth Inhibition

Growth inhibitory effects of chemotherapy and supplements were evaluated on MTS [3-(4,5-dimethylthiazol-2-yl)-5-(3-carboxymethoxyphenyl)-2-(4-sulfophenyl)-2H-tetrazolium] assay, which measures mitochondrial activity of viable cells. Lung cancer cells were plated onto flat bottom 96-well plates and allowed to attach for 24 h. The cells were treated for different time periods. After treatment, the medium was removed and the cells were incubated for 45 min at 37°C in MTS solution (MTS : PMS = 20 : 1) with a total volume of 120 *μ*L per well. The absorbance was measured at 495 nm using an ELISA reader. The results are presented as the mean ± SD. of quadruplicate experiments repeated at least three times.

### 2.4. Cell Cycle and Apoptosis Analyses

Cells (3 × 10^5^ cells) were plated onto 10 cm dish and allowed to attach for 24 h prior to drug treatment. The exposure time ranged from 4 to 5 days for single drug or supplement combination. The adherent and floating cells were harvested and counted. After centrifugation, the cells were fixed with 1 mL ice-cold 75% ethanol overnight. Subsequently, the cells were collected by centrifugation, and the cell pellets were treated with 30 *μ*L RNase A (10 mg/mL) and PBS (300 *μ*L) for 30 min. Cells were then gently resuspended in 10 *μ*L propidium iodide (500 *μ*g/mL) with PBS (300 *μ*L) and stored on ice for 30 min. Cell cycle analyses were performed using a FACScan (BD Biosciences, Mount View, CA, USA). Data analysis was carried out with CELLQuest software, using gates on DNA histograms to estimate the quantities of cells in G1, S, and G2/M phases, as well as the quantity of apoptotic cells in sub-G1 region.

### 2.5. Western Blot Analysis

Lung cancer cells were resuspended in 1x PBS reagent in the presence of a protease inhibitor cocktail (Sigma-Aldrich). Protein content was assessed using the Bio-Rad assay (Bio-Rad, Hercules, CA, USA) and analyzed by SDS-PAGE. Equal amounts of total protein were separated on 10–15% SDS-PAGE and transferred to PVDF membranes. After blocking with 3% nonfat dry milk/Tris-buffered saline, the membranes were incubated overnight at 4°C with primary antibodies. The following antibodies were used: *β*-actin (1/500, AC-40, Sigma-Aldrich), antithymidylate synthase (1/500, Santa Cruz, CA, USA), anti-p53 (1/2000, Dako Corporation, Carpinteria, CA, USA), and anti-p21^WAF1/Cip1^ (1/500, Zymed, Invitrogen, Carlsbad, CA, USA). On the following day, the membranes were incubated with the appropriate horseradish peroxidase-conjugated secondary antibodies (Calbiochem, San Diego, CA, USA), and detection was performed using E.C.L (GE Healthcare Bioscience, Amersham Place, UK). Image J quantity software was downloaded from the National Institutes of Health and used to measure the intensity of each blot.

### 2.6. Statistical Analysis

All values are expressed as mean ± SD from at least three independent experiments. Student's *t*-test was performed for comparison of data from independent samples. A probability (*P*) value <0.05 was considered significant.

## 3. Results

### 3.1. High Level of Fetal Bovine Serum (FBS) Reduces the Sensitivity of Human NSCLC Cells to MTA Treatment

**Figure 1:**
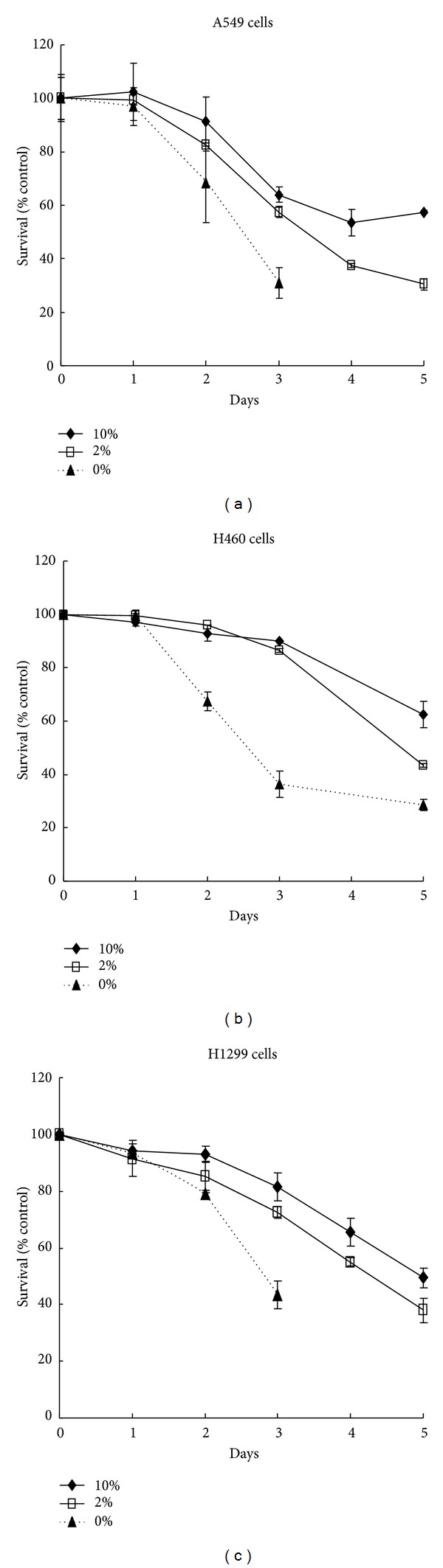
Effects of fetal bovine serum (FBS) on MTA sensitivity in human NSCLC cells. Cell cytotoxicity (MTS) assay was performed to measure the sensitivity of cells to MTA (500 nM) under different levels of FBS in culture medium. (a) A549 (400 cells/well), (b) H460 (10^3^ cells/well), and (c) H1299 cells (10^3^ cells/well) were seeded onto 96-well plate for 24 h with 10%, 2%, or 0% (serum-free) serum for 5 days. Paired samples were prepared at each time point, and the survival was calculated by normalizing to the sample without MTA treatment, represented as 100%. The reduced values of survival (% control) indicated the sensitivity of cells to MTA treatment.

As MTA is an antifolate, it competes with natural sources of folates inside and outside the cell. Gates et al. have shown that changes in dietary folate intake can modulate antifolate efficacy in mice [15]. Therefore, we attempted to determine whether the efficacy of MTA is reduced in cancer cells maintained under normal nutrient conditions. Three NSCLC cell lines were used to test the nutrition effect of serum on MTA drug efficacy. The sensitivities of A549 (Figure 1(a)), H460 (Figure 1(b)), and H1299 (Figure 1(c)) cells were analyzed with 500 nM MTA under 10% and 2% serum conditions. Increase in serum level from 2 to 10% resulted in decreased sensitivity to MTA. Under 10% serum conditions, the efficacy of MTA was significantly reduced in all three tested cell lines. Cancer cells grown for several days without serum were prone to death. However, on day 2, the MTA sensitivity was higher in serum-free cells than in cells grown in 2% or 10% serum. On day 5, the sensitivities to MTA with 2% serum were higher than those with 10% serum in all three cell lines. The RPMI medium contains high levels of FA (2.2 *μ*M) and VB12 (3.7 nM). Thus, cells should have been maintained without FA/VB12 deficiency. The results imply that sensitivity of cancer cells to MTA is reversely associated with serum factors other than FA/VB12.

### 3.2. Addition of FA and VB12 Enhances the Sensitivity of NSCLC Cells to MTA Treatment with Regular Nutrient Conditions

**Figure 2:**
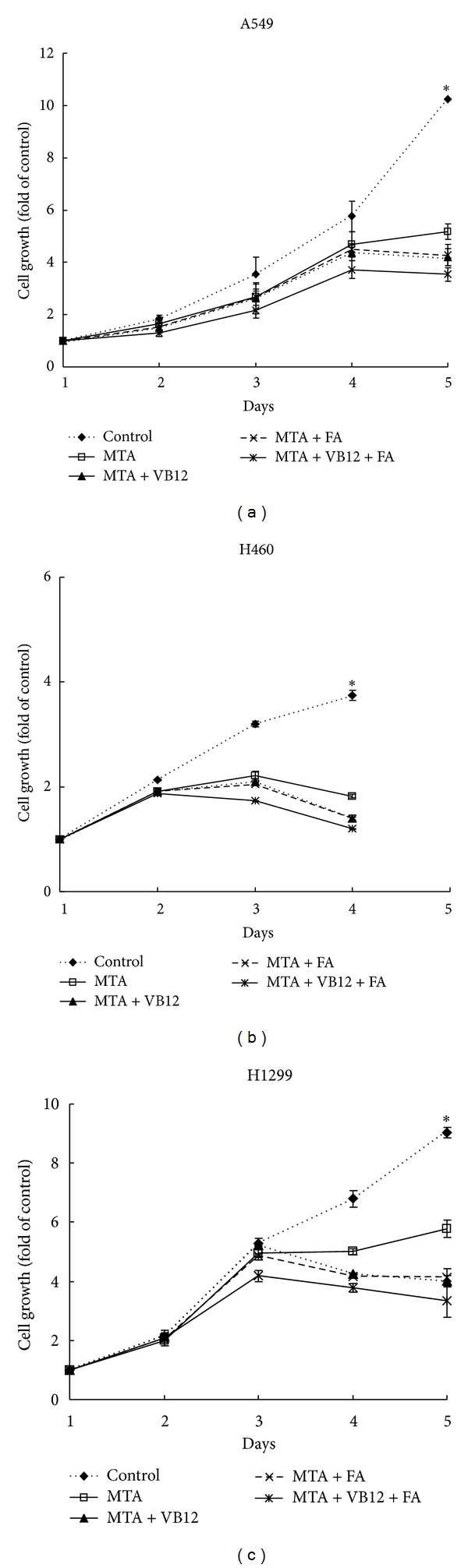
Effects of FA/VB12 supplementation on MTA-treated NSCLC cells with 10% serum. Five sets of cells were analyzed on MTS assay to determine cell growth. (a) A549 (400 cells/well), (b) H460 (10^3^ cells/well), and (c) H1299 cells (10^3^ cells/well) were seeded onto 96-well plate for 24 h on day 1 with 10% serum followed by MTA and FA/VB12 treatment. Cells without MTA treatment served as the control (control). The cells were maintained in 10% serum combined with MTA (500 nM, MTA), MTA with 600 nM FA (MTA + FA), MTA with 600 nM VB12 (MTA + VB12), or MTA with FA and VB12 (MTA + FA + VB12) for 5 days. The cell growth ratio was normalized to day 1 of each set of cells. The *P* value (* < 0.05) was determined by Student's *t*-test.

Since patients treated with MTA receive high doses of FA and VB12, they are assumed to maintain normal or high serum levels of FA and VB12 without deficiency. Therefore, we evaluated the possible antagonistic effect of FA and VB12 on MTA drug efficacy with 10% serum to reflect *in vivo* nutrient conditions. A549 cells were maintained in 10% serum with MTA (500 nM), MTA and FA (600 nM), MTA and VB12 (600 nM), or MTA, FA and VB12 for five days (Figure 2(a)). The cells without MTA treatment served as the control. The cells treated with MTA alone showed reduced growth rate when compared with the control cells. On day 5, the number of MTA-treated cells was 5.2-fold higher than that on day 1. In contrast, the number of cells without MTA treatment was 10.2-fold higher than with MTA treatment on day 1. This result demonstrated that MTA treatment inhibits cells with 51% of cells remaining on day 5 when compared with control. Addition of FA or VB12 to MTA-treated cells had no apparent antagonistic effect after three days when compared with the cells treated with MTA alone. Interestingly, on day 5, the FA-supplemented and VB12-supplemented groups showed higher sensitivity to MTA treatment. The results demonstrated 41% of remaining cells in groups supplemented with VB12 or FA when compared with control. Moreover, when FA and VB12 were combined with MTA, the sensitivity was significantly enhanced so that only 34% of cells survived when compared with control (P* < 0.05). To confirm whether this effect is also present in other lung cancer cell lines, H460 and H1299 cells were grown under the same conditions. We found significantly enhanced sensitivity in H460 (Figure 2(b)) and H1299 cells (Figure 2(c)) cultured with FA and VB12 under 10% serum conditions on day-5. In H460 cells, when compared with the control, MTA treatment inhibited cells with 49% of cells remaining. FA and VB12 supplementation resulted in 32% surviving cells (P* < 0.05). In H1299 cells, MTA treatment resulted in 64% surviving cells. There were 37% surviving cells with MTA, FA, and VB12 combined treatment (P* < 0.05). Therefore, no antagonistic effect of FA/VB12 on MTA was detected in lung cancer cells maintained under normal culture conditions. Furthermore, our data suggested that when lung cancer cells are grown under normal nutrient conditions, MTA efficacy is enhanced by FA and VB12.

### 3.3. FA and VB12 Have Additive Effect to Enhance the MTA Drug Efficacy

**Figure 3:**
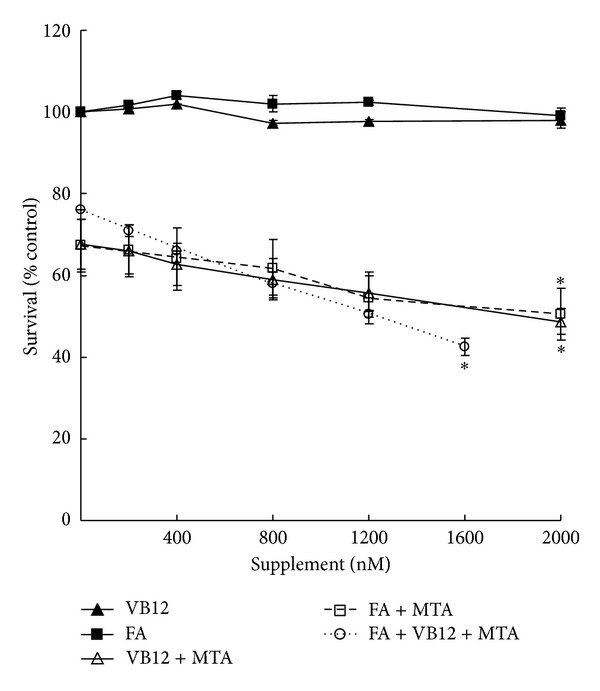
Correlations of FA and VB12 concentrations with MTA efficacy. A549 cells were seeded onto 96-well plate with 10% serum followed by MTA and FA/VB12 treatment. On day 5, the remaining cells were measured by MTS assay. The possible toxic effects of FA and VB12 (400 to 2,000 nM) without MTA treatment were determined. The experimental sets were FA combined with MTA (FA + MTA) and VB12 combined with MTA (VB12 + MTA). The additive effect of FA and VB12 on MTA efficacy was measured. FA and VB12 in the same concentrations in each combination were applied to measure their effects on MTA efficacy (FA + VB12 + MTA). The *P* value (* < 0.05) was determined by Student's *t*-test.

Our data showed that FA and VB12 have similar positive effect on MTA drug efficacy. When added together, they further enhance drug efficacy. Therefore, we examined the dosage effect of FA and VB12 on MTA drug efficacy. A549 cells were treated with various concentrations of FA or VB12 ranging from 400 to 2,000 nM without MTA for five days as the control to test for possible toxicity. Although the RPMI medium and 10% BSA already contain high levels of FA and VB12, no apparent toxicity was observed even at 2,000 nM FA or VB12 (Figure 3). When increasing amounts of FA were combined with MTA (500 nM) for 5 days of treatment, the number of surviving cells decreased in a dose-dependent manner. The survival ratio was 67% for cells treated with MTA alone and decreased to 51% in cells cotreated with MTA and FA (2,000 nM). Similar effect was also detected in cells cotreated with MTA and VB12 (2,000 nM). The MTA efficacy increased approximately to 16% (P* < 0.05) under cotreatment with FA (2,000 nM) or VB12 (2,000 nM). When FA and VB12 in the same concentrations (1,600 nM) were combined with MTA treatment, MTA efficacy increased to approximately 32% (P* < 0.05). Overall, these results demonstrated that FA and VB12 individually enhance MTA drug efficacy. When acting together, FA and VB12 have an additive effect on cell sensitivity to MTA under 10% serum conditions. Again, these data revealed that FA and VB12 supplementation has no antagonistic effect on MTA efficacy.

### 3.4. FA and VB12 Enhance the MTA but Not the 5-FU Drug Efficacy

**Figure 4:**
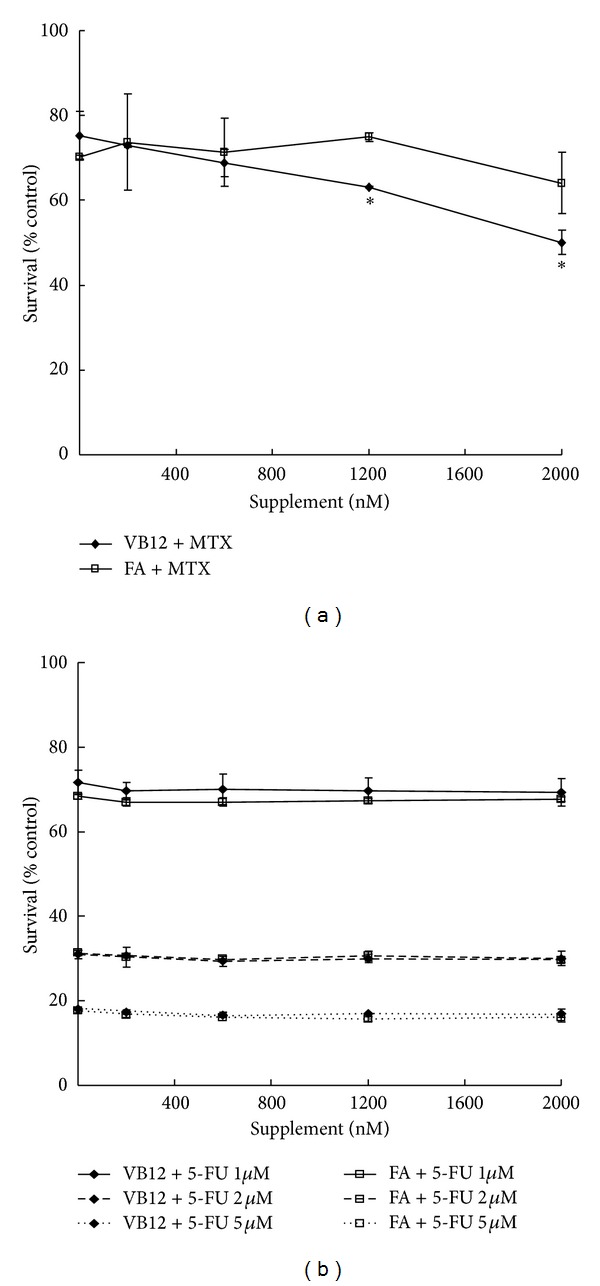
Effects of FA and VB12 on other antimetabolites. Drug sensitivity was analyzed by MTS assay to determine cell growth. (a) The possible effects of FA (FA + MTX) and VB12 (VB12 + MTX) on MTX (800 nM) treatment were measured on day 5. (b) The possible effects of FA (FA + 5-FU) and VB12 (VB12 + 5-FU) on 5-FU (1, 2, 5 *μ*M) treatment were measured on day 4. The *P* value (* < 0.05) was determined by Student's *t*-test.

The effects of FA and VB12 on the efficacy of other antimetabolites were examined. The classic antifolate methotrexate (MTX) is a DHFR inhibitor [16] that shares antimetabolic pathways with MTA [17]. Hence, we examined whether addition of FA and VB12 affects MTX efficacy. A549 cells were treated with 800 nM MTX combined with various concentrations of FA or VB12 ranging from 400 to 2,400 nM (Figure 4(a)). VB12 significantly enhanced MTX efficacy (P* < 0.05) at higher dosages (Figure 4(a), 1,200 and 2,000 nM). MTX drug efficacy was also enhanced with higher dosages of FA, but the difference was not statistically significant. We used low to high-doses of 5-FU, another TS inhibitor of antimetabolite, to treat A549 cells. If FA and VB12 are antagonists of 5-FU, under high dose 5-FU conditions, drug efficacy may be reduced. In contrast, enhancement may be observed under low dose 5-FU conditions. Different doses of 5-FU (1, 3, 5 *μ*M) resulted in different percentages of surviving cells on the first day of treatment. Interestingly, the efficacy of 5-FU was not affected by addition of varying concentrations of VB12 or FA in A549 cells treated with different doses of 5-FU (Figure 4(b)). Supplementation with FA or VB12 has no significant effect on 5-FU efficacy.

### 3.5. FA and VB12 Supplementation Increases Apoptosis of Cancer Cells with MTA Treatment

To further characterize the effect of FA/VB12 supplementation on MTA drug efficacy, the sub-G1 phase of cells was analyzed by flow cytometry.  Table 1 presents the results from the analyzed A549, H460, and H1299 cells. MTA alone induced 4.58% sub-G1 population in A549 cells. When FA and VB12 were combined with MTA, the sub-G1 population increased to 7.21% (P* < 0.05) in A549 cells. Similar increases in sub-G1 populations in H460 and H1299 cells were demonstrated. FA or VB12 alone increased the sub-G1 populations in tested cells but not as dramatically as FA and VB12 in combination. Logically, a high dose of FA/VB12 supplementation in MTA-treated patients not only reduces the cytotoxicity resulting from antifolate chemotherapy, but also induces more cancer cells to undergo apoptosis. 

### 3.6. MTA Regulates TS, p53, and p21 Expressions and FA/VB12 Stabilizes p53 and p21 Expressions

**Figure 5:**
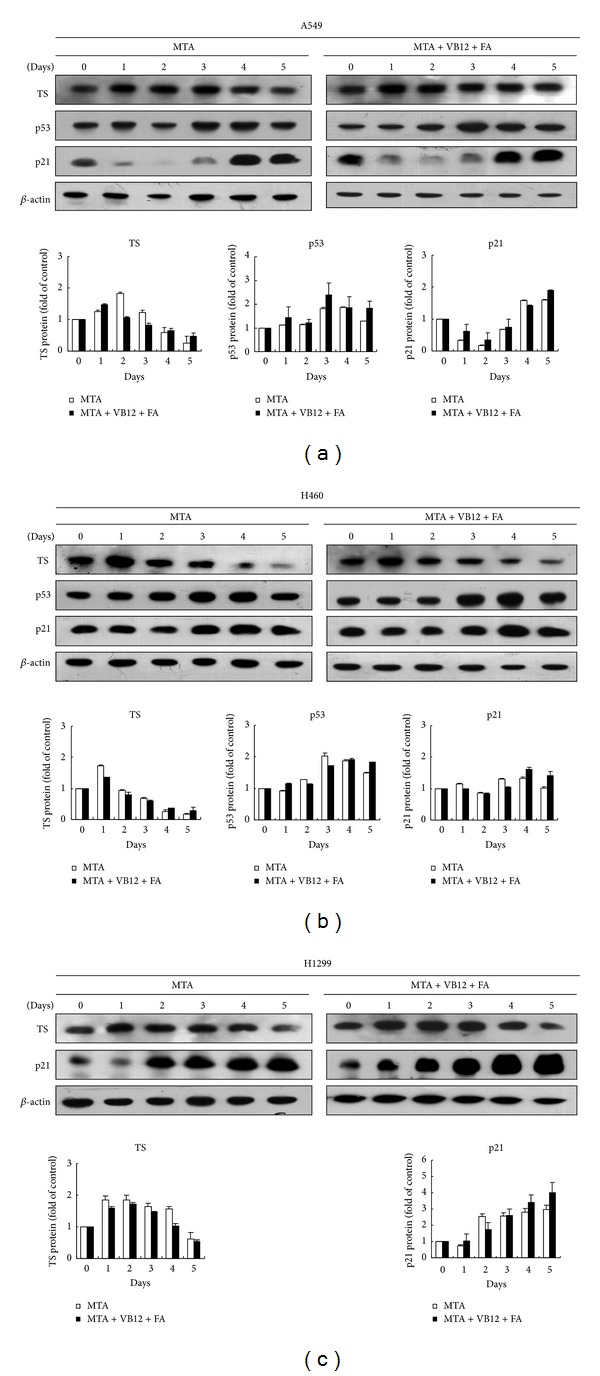
Western blot analysis of the MTA-regulated proteins in A549, H460, and H1299 cells. (a) A549, (b) H460, and (c) H1299 cells (10^5^ cells) were seeded onto 6 cm dishes and allowed to attach for 24 h prior to drug treatment (day 0). MTA (500 nM), FA (1,600 nM), and VB12 (1,600 nM) were added with fresh medium, and cells were harvested at the indicated times. The proteins of TS, p53, p21^WAF1/Cip1^, and *β*-actin were determined by immunoblot experiments with the corresponding antibodies. Expression of *β*-actin on the same immunoblot was used as a loading control.

To examine the mediators that cause apoptosis increase in cells cotreated with FA, VB12 and MTA, the cell cycle inhibitors of p53 and p21^WAF1/Cip1^ proteins were analyzed on western blot. The TS protein levels increased significantly, reaching maximal level on day 2 of MTA treatment, and then diminished (Figure 5(a)). The expression patterns of TS were similar in cells treated with MTA (500 nM) alone and cells treated with MTA and FA/VB12 (1,600 nM each). The data further demonstrated no antagonistic effect from FA/VB12 supplementation. The expression levels of p53 protein were affected by MTA treatment in A549 cells. MTA dramatically stimulated the expression of p53 protein on day 3 and this elevation in p53 protein level was sustained thereafter. Interestingly, the expression levels of p21 were markedly inhibited upon MTA treatment and recovered on day 4 of MTA treatment (Figure 5(a)). The expressions of TS, p53, and p21 in H460 cells showed similar patterns when compared with A549 cells (Figure 5(b)). The p53-null H1299 cells also showed upregulated TS levels initially. These levels were downregulated on day 4 of MTA treatment (Figure 5(c)). The levels of p21 protein significantly increased in H1299 cells treated with MTA. When the levels of p53 and p21 proteins were calibrated with *β*-actin and normalized to day 0, the results showed that the levels of p53 and p21 proteins were sustained on day 5 in FA/VB12 supplemented cells treated with MTA when compared with cells treated with MTA alone.

## 4. Discussion

High doses of FA and VB12 in patients with malignant pleural mesothelioma have been successful in reducing MTA toxicities [13]. However, the question of whether vitamin supplementation affects MTA's efficacy remains. It has been reported that increased level of natural folates results in diminished activity of antifolate drugs in murine leukemia cells [18]. By examining normal and neoplastic murine tissues and human tumor xenografts, dietary folate intake significantly modulates FPGS activity *in vivo *[15]. Thus, the cellular pool of folates affects antifolate activities. In our experiments, cancer cells were maintained in growth conditions without natural folates and VB12 deficiencies. It is possible that poor nutrition affects cells under low serum conditions resulting in higher sensitivity to MTA. Our original hypothesis was that the addition of high doses of FA and VB12 reduces MTA activity in the tested cell lines. Surprisingly, MTA activity was enhanced by FA and VB12 supplementation in cells under 10% serum conditions. This effect was also observed in H460 and H1299 cells (Figure 2). To rule out the possibility of cytotoxicity of FA/VB12 contributing to MTA toxicity, we treated the cells with very high doses of FA or VB12 (up to 2,000 nM) without MTA in 10% serum. No apparent toxicity was observed. When higher doses of FA and VB12 were applied to the cells treated with MTA, efficacy was further enhanced (Figure 3). Our results showed no antagonistic effect by FA/VB12 on MTA under cell culture conditions. Supplementation not only ameliorates toxicity without reducing MTA efficacy, but also enhances MTA efficacy. Our data may partially explain why malignant pleural mesothelioma patients supplemented with FA/VB12 tolerate treatment better (less toxicity and more cycles of treatment) and have a higher 5-month median overall survival than nonsupplemented patients [19]. Therefore, FA/B12 status and other nutrition factors could be considered as two independent factors that are associated with MTA sensitivity. Since the efficacy could be enhanced with low-nutrition conditions, thus, toxicity increased and the lethality may be increased in low-nutrition conditions, even with FA/VB12 supplementation in pemetrexed therapy.

It has been reported that MTA has additive effects with 5-FU [20], while synergistic effects have been noted in combination with gemcitabine [21]. To further investigate the specificity of supplementation effects, A549 cells were treated with 5-FU. FA/VB12 supplementation showed no apparent effect (Figure 4(b)). Although TS is the target of 5-FU, but 5-FU is not the substrate of FPGS for polyglutamation of folate. The concentration of 5-FU determining its efficacy without being affected by FA/VB12. Therefore, we found that there was no effect of FA/VB12 on 5-FU efficacy. MTX is an antifolate drug that is used to treat rheumatoid arthritis [22] and is particularly important in the treatment of childhood leukemias [23]. The advantages and disadvantages of FA supplementation in rheumatoid arthritis therapy with MTX have been discussed in detail [24, 25]. According to our data, FA has no antagonistic effect, while VB12 significantly enhances effect of MTX on lung cancer cells. The possible explanation could be that DHFR is the target of MTX that is acting downstream of TS in folate metabolism. High folate level may have no effect on DHFR, but the downstream level of VB12 may affect DHFR to enhance MTX efficacy. Hence, FA has no effect, while VB12 significantly enhances MTX efficacy in lung cancer cells. These results indicated that FA/VB supplementation specifically enhances MTA efficacy. Further investigation is needed to determine if other antifolate drug activities are affected.

To demonstrate whether FA and VB12 contribute a sensitizing effect to MTA-treated cancer cells that, in turn, increases the efficacy of MTA therapy, we analyzed cell cycle distribution and apoptosis using flow cytometry. The results were similar to previously reported data [26] in which S phase arrest and induction of apoptosis markedly increase after MTA treatment. In contrast to MTA alone, FA/VB12 supplementation significantly increased sub-G1 population in human adenocarcinoma cell line A549 and large cell carcinoma cell lines H1299 and H460. The p53 statuses of A549 (wt-p53) and H1299 (null-p53) were different, but the MTA sensitivity was enhanced by FA/VB12 supplementation in both cell lines. According to the results of this study, effect of FA/VB12 supplementation is independent of p53 status.

The expression of TS, one of the targets of MTA, is considered a major determinant of antifolate sensitivity [17]. Downregulation of TS expression was demonstrated in A549, H460, and H1299 cells after MTA treatment. Interestingly, TS protein levels were upregulated when cancer cells were exposed to MTA, reaching maximal level, and then diminished to below basal level (Figure 5). Upregulation of TS expression in the initial phase of antifolate exposure has also been demonstrated in W1L2 (human lymphoblastoid) and A549 cells treated for 24 h [27]. TS protein transiently increases to respond to MTA inhibition effect. When TS protein diminishes, cytotoxicity occurs as illustrated by increased sub-G1 population.

We also showed that p53 protein is upregulated one day after TS protein increases and persists above basal level. In contrast to TS, our data showed that p21^WAF1/Cip1^ protein is downregulated when TS is upregulated. In addition, when TS protein level gradually decreased, p21 protein level increased. Taken together, the reverse correlation of TS and p21 expression is not associated with p53 status of cells.

The effect of high doses of FA/VB12 supplementation was not only supported by our data of increased apoptosis of sub-G1 population but also by sustained p53 and p21 levels. The critical role of p21 has been demonstrated in human colorectal cancer cells treated with another TS inhibitor (ZD9331) [28]. Therefore, MTA may regulate p21 expression through p53-dependent and -independent pathways in cancer cells.

In conclusion, we demonstrated that high dose FA/VB12 supplementation is not antagonistic to MTA activity *in vitro*. Increased drug efficacy is specific to MTA and is not associated with p53 status of the cells. In addition, the enhanced effect may be positively correlated with p21^WAF1/Cip1^ expression. Since cancer patients are usually supplemented with vitamins without prescription, our data could relieve the concern of antagonism to MTA therapy. We strongly suggest that more *in vitro* and human studies will be necessary to verify whether high dose FA/VB12 supplementation is not antagonistic to MTA activity in clinical applications.

## Figures and Tables

**Table 1:** Sub-G1 analyses of A549, H460, and H1299 cells by flow cytometry.

% of sub-G1					
Cell line	Control	MTA	MTA + VB12	MTA + FA	MTA + VB12 + FA
A549	0.26 ± 0.13	4.58 ± 0.56	6.15 ± 1.17	5.54 ± 1.47	7.21 ± 0.17*
H460	0.21 ± 0.05	9.81 ± 0.91	14.51 ± 1.57*	15.7 ± 4.02*	19.28 ± 1.03*
H1299	0.28 ± 0.06	23.43 ± 2.67	28.97 ± 2.29*	30.62 ± 0.15*	36.75 ± 3.69*

*MTA: Pemetrexed, VB12: vitamin B_12_, and FA: folic acid.

As described in Section 2, cells were treated with MTA (500 nM), MTA and VB12 (600 nM), MTA and FA (600 nM), or MTA with VB12 and FA (600 nM each). On day 4, H460 cells were collected and analyzed. On day 5, A549 and H1299 cells were analyzed by flow cytometry. The *P* value (* < 0.05) was determined by Student's *t*-test.
